# Long-Acting Injectable Antipsychotics in First-Episode Schizophrenia

**DOI:** 10.1155/2012/318535

**Published:** 2012-08-21

**Authors:** Eduard Parellada, Dawn I. Velligan, Robin Emsley, Werner Kissling

**Affiliations:** ^1^Clinic Schizophrenia Program (PEC), Department of Psychiatry, Hospital Clinic of Barcelona, University of Barcelona, 08036 Barcelona, Catalonia, Spain; ^2^Department of Psychiatry, University of Texas Health Science Center San Antonio, 7300 Floyd Curl Drive, San Antonio, TX 78229, USA; ^3^Department of Psychiatry, Faculty of Medicine and Health Sciences, Stellenbosch University, Tygerberg, Cape Town 7500, South Africa; ^4^Klinik und Poliklinic für Psychiatrie und Psychotherapie, Klinikum Rechts der Isar, Technische Universitaet München, Moehlstraße 26, 81675 München, Germany

Long-acting injectable antipsychotics (LAIAs) may improve adherence to treatment and reduce the rate of relapse and rehospitalization in first-episode or recent-onset schizophrenia (e.g., less than 2 years of illness duration). However, despite their potential advantages, LAIAs are underutilised in clinical practice and the place of LAIAs in the early phases of schizophrenia is still a controversial clinical issue. For example, negative attitudes toward LAIAs in first-episode schizophrenia among psychiatrists are common, and the place of LAIAs for first-episode psychoses (FEPs) remains uncertain in the current clinical guidelines for the pharmacological treatment of schizophrenia. Moreover, a recent paper published in the New England Journal of Medicine by Rosenheck et al. [1] reported negative results of LAI risperidone (RLAI) on relapse prevention, although this was in a multiepisode sample. The recent and forthcoming availability of additional second-generation LAIAs (SG-LAIAs), namely, olanzapine pamoate, paliperidone palmitate, aripiprazole, and iloperidone depot, will add interest to this clinical debate for practicing clinicians and researchers interested in this timely topic.

This special issue seeks to define the place of LAIAs in the treatment of first-episode or recent-onset schizophrenia.

S. Zhornitsky and E. Stip present a systematic review examining the efficacy and tolerability of LAIAs versus their oral equivalents in randomized and naturalistic studies. In addition, they examine the impact of LAIAs on special populations at risk for treatment nonadherence such as patients with FEP, substance use disorders, and a history of violence or on involuntary outpatient commitment. Randomized studies suggest that not all LAIAs are the same in terms of side effects. They also suggest that LAIAs reduce risk of relapse versus oral antipsychotics in schizophrenia outpatients when combined with quality psychosocial interventions. Finally, large-scale naturalistic studies point to a larger magnitude of benefit for LAIAs, relative to their oral equivalent antipsychotics, especially among FEP patients.

One of the original studies (by A. Viala et al.) reports a naturalistic, open-label study of 25 patients in the early phases of schizophrenia treated with RLAI and followed up at least 18 months. The authors found that patients receiving RLAI had a favourable global outcome.

As already mentioned, although not found in all studies [1], there is growing evidence that the risk of relapse is lower with LAIA versus oral antipsychotics. In this sense, two recent studies published in 2011 deserve to be emphasized. First, a recent meta-analysis published by Leucht et al. [2] including all randomized controlled trials (RCTs) comparing LAIAs with oral formulations showed a reduced risk for relapse associated with LAIAs over oral antipsychotics. Second, the Tiihonen et al. [3] cohort study of oral and depot antipsychotics after first hospitalization for schizophrenia in 2,588 FEP patients found that fewer than 50% of patients in the Finnish health care system continue treatment for the first 2 months after an initial hospitalization for schizophrenia. In this study, route of treatment administration also affected relapse rate. LAIAs had a 64% lower relapse rate than equivalent oral medication.

The paper published by R. Přikryl et al. in this special issue reviews the role of SG-LAIAs in treatment of first-episode schizophrenia patients and argues in favour of the use of LAIAs very convincingly in terms of clinical judgment. This paper also focuses on negative attitudes toward injectable medications among psychiatrists being one of the barriers that may explain the underutilization of LAIAs, especially in FEP patients, as reported by Heres et al. [4]. Other barriers to the use of LAIAs (negative attitudes among patients, reimbursement and logistical issues, etc.) should also be addressed. In this sense, some recent strategies for initiating a long-acting injection clinic in public health care centres and initiatives to provide education to prescribers and patients deserve mention: the ShoT At Recovery (A-STAR) program [5], the Munich Compliance Program, and the CERP Program [6]. The former is a LAIA program developed in Texas, based on a multidisciplinary treatment team to support adherence and recovery for patients on LAIAs. The Centres of Excellence for Relapse Prevention (CERP) in Schizophrenia Program is an international educational activity initiated by an international group of expert psychiatrists to address the worldwide issue of relapse among patients with psychotic disorders, especially schizophrenia. It is a new forum for education and information sharing around the topic of relapse and relapse prevention strategies including the early stages of illness.

One additional paper (see B. Kim et al.) reviews clinical trials, survey studies, and current international guidelines on the use of LAIAs in first-episode schizophrenia and considers the pros and cons of this treatment option. The paper presents a brief overview of a few preliminary naturalistic and randomized clinical studies primarily designed to evaluate SG-LAIAs in first-episode schizophrenia. Published clinical guidelines reflect uncertainties in the use of LAIAs in the critical early period of the illness. With some exceptions, the majority of treatment guidelines limit the use of LAIAs to multiple-episode patients and to openly nonadherent patients [7]. Clearly, the current clinical guidelines regarding LAIAs use are too conservative.

The objective of the original research paper published by Ch. Asseburg et al. is to quantify changes in hospital resource use in a naturalistic clinical setting in schizophrenia patients in Finland following initiation of RLAI. Although not primarily focusing on FEP, the study found that consistent reductions in resource use are associated with the initiation to RLAI in Finland. These results agree with several recent studies exploring the issue of health resource utilization and cost-effectiveness [8, 9].

Finally, we would like to outline three unmet research needs concerning LAIAs in FEP for the future.

First, there is a need for better designed RCTs in FEP. There is an absence of long-term RCTs comparing LAIAs with oral medication after FEP regarding efficacy, tolerability, relapse prevention, and global outcomes. We also need studies examining patient preferences, acceptability, and attitudes toward LAIAs in early phases of the illness, as well as data about nonadherence rates of SG-LAIAs in early phases of the schizophrenia. There is also a lack of cost-effectiveness studies comparing LAIAs with oral antipsychotic treatments specifically focusing on first-episode schizophrenia patients.

Second, the question of whether effective early intervention positively influences long-term outcome needs to be more effectively addressed. We need to know whether we are able to alter disease trajectory to clinical and neurological deterioration that mainly occurs within the first 3–5 years following the onset of the illness. Although there is some evidence to suggest a better global outcome using LAIAs as compared to oral antipsychotics with a reduced risk of relapse and rehospitalization [10, 11], it is still not clear whether these agents can improve biological and clinical outcomes by reducing early relapse and loss of function in first-onset patients. A positive answer for benefits on disease progression would provide support to an emerging literature regarding the neuroprotective effects of the antipsychotics, especially SG antipsychotics [12–14].

Third, we need increased availability of additional SG-LAIAs and to develop more reliable methods of antipsychotic delivery. Given the failure of the long-term oral treatments and keeping in mind that relapse can lead to serious consequences from all perspectives (biological and psychosocial), the future of the schizophrenia pharmacotherapy will hopefully evolve to include better long-term delivery systems such as longer extended release injectable formulations, transdermal patches, subcutaneous implants of antipsychotics, and other long-acting devices like antipsychotic pumps to more effectively address the high risk of relapse due to nonadherence early in the course of illness. Antipsychotic release of skin implants containing risperidone and biodegradable polymers has been already assessed in vitro and in vivo in animal models [15, 16]. Such devices could however raise concerns regarding the therapeutic alliance and obvious issues of medical ethics that should be appropriately addressed.

To summarize, considering that poor adherence to oral antipsychotic treatments and the very high relapse rates early in the illness due to nonadherence are the rule rather than an exception, from the clinical point of view, psychiatrists should think in terms of relapse prevention from the outset of the illness, identify and overcome local barriers to use LAIAs, and consider the option of SG-LAIAs to all patients with first-episode or recent-onset schizophrenia in a shared decision-making approach. The success of such pharmacological intervention would of course be enhanced by combining with appropriate psychosocial interventions within a relapse prevention program. In this sense, the current clinical guidelines regarding LAIA use in FEP are much too conservative and need to be updated. However, it needs to be remembered that there is still a need for more open-label or double-blind RCTs in early phases of schizophrenia, regarding the long-term efficacy, safety, global functional outcome, and cost-effectiveness of SG-LAIAs compared to oral antipsychotics in order to obtain a more robust clinical database for evidence-based medicine. Such studies will also define whether or not LAIAs introduced early in the course of the schizophrenia illness can alter the disease trajectory.
